# Mammalian Target of Rapamycin (mTOR) Activity Dependent Phospho-Protein Expression in Childhood Acute Lymphoblastic Leukemia (ALL)

**DOI:** 10.1371/journal.pone.0059335

**Published:** 2013-04-03

**Authors:** Karolina Nemes, Anna Sebestyén, Ágnes Márk, Melinda Hajdu, István Kenessey, Tamás Sticz, Eszter Nagy, Gábor Barna, Zsófia Váradi, Gábor Kovács, László Kopper, Monika Csóka

**Affiliations:** 1 2^nd^ Department of Pediatrics, Semmelweis University, Budapest, Hungary; 2 1^st^ Department of Pathology and Experimental Cancer Research, Semmelweis University, Budapest, Hungary; 3 Tumor Progression Research Group of the Hungarian Academy of Sciences and Semmelweis University, Budapest, Hungary; 4 2^nd^ Department of Pathology, Semmelweis University, Budapest, Hungary; 5 National Institute of Rheumatology and Physiotherapy, Budapest, Hungary; Westmead Millennium Institute, University of Sydney, Australia

## Abstract

Modern treatment strategies have improved the prognosis of childhood ALL; however, treatment still fails in 25–30% of patients. Further improvement of treatment may depend on the development of targeted therapies. mTOR kinase, a central mediator of several signaling pathways, has recently attracted remarkable attention as a potential target in pediatric ALL. However, limited data exists about the activity of mTOR. In the present study, the amount of mTOR activity dependent phospho-proteins was characterized by ELISA in human leukemia cell lines and in lymphoblasts from childhood ALL patients (n = 49). Expression was measured before and during chemotherapy and at relapses. Leukemia cell lines exhibited increased mTOR activity, indicated by phospho-S6 ribosomal protein (p-S6) and phosphorylated eukaryotic initiation factor 4E binding protein (p-4EBP1). Elevated p-4EBP1 protein levels were detected in ALL samples at diagnosis; efficacy of chemotherapy was followed by the decrease of mTOR activity dependent protein phosphorylation. Optical density (OD) for p-4EBP1 (ELISA) was significantly higher in patients with poor prognosis at diagnosis, and in the samples of relapsed patients. Our results suggest that measuring mTOR activity related phospho-proteins such as p-4EBP1 by ELISA may help to identify patients with poor prognosis before treatment, and to detect early relapses. Determining mTOR activity in leukemic cells may also be a useful tool for selecting patients who may benefit from future mTOR inhibitor treatments.

## Introduction

Acute lymphoblastic leukemia (ALL) is the most common malignant disease diagnosed in children, representing nearly one third of all pediatric malignancies. The annual incidence of acute lymphoblastic leukemia is approximately 4–5 cases per 100 000 of the childhood population, with a pjavascript:showcontent (‘active’, ‘references’); eak incidence in children aged between 1–6 years [1], [2]. Modern treatment strategies consisting of intensive chemotherapy, cranial and/or testicular irradiation and stem cell transplantation have remarkably improved the prognosis of childhood ALL [3]. However, patients with good survival chances are at risk of severe acute and late adverse effects of therapy [4], and treatment still fails in 25–30% of patients. Targeted therapy is one of the possibilities to improve survival rates [5], [6].

Several signal transduction pathways (PI3K/AKT/mTOR, JAK/STAT, ABL tyrosine kinase, SRC family of tyrosine kinases and NOTCH1) play a role in normal B- and T-cell development, proliferation, survival and activation [5]. Deregulation of these networks is likely to be a key event in leukemogenesis [7]. Recently, mTOR (mammalian target of rapamycin) has gained remarkable attention as a potential target in different tumor types, including hematological malignancies [8], [9]. The mTOR serine-threonine kinase is a central mediator of several signal transduction pathways, and it is considered a regulator of cell proliferation, protein translation and survival [10], [11]. mTOR is able to form two distinct complexes (mTORC1 and mTORC2) [12], which can be activated by various growth factors, cytokines and nutrients. The mTORC1 complex is sensitive to currently used mTOR inhibitors (MTIs: rapamycin and rapalogs). Activated mTORC1 phosphorylates two key translational regulators: eukaryotic initiation factor 4E binding protein (4EBP1) [13] and 70 kDa S6 ribosomal protein kinase (p70S6K) [14]. mTORC2 works in concert with PDK1 to phosphorylate/activate AKT; however, the function and regulation of mTORC2 and its response to rapamycin remains unclear, and may vary in different cell types [15], [16].

Mechanisms which contribute to increased mTOR activity in ALL may be the activation of PI3K/AKT by mutations of the tumor suppressor gene PTEN [17], and by abberant signals from TCL1, BCR-ABL, growth factor receptors (such as IGF-1R and c-kit), IL-7R, flt-3 [18], [19], [20], [21], [22] and oncogenic NOTCH1, the latter one specifically in T-ALL. Inactivation of the FBW7 ubiquitin ligase – which is important for the degradation of mTOR – was also found in 20% of T-ALLs [23].

Anti-neoplastic properties of MTIs were noted shortly after their discovery, nevertheless, they were most widely used as immunosuppressive agents for a long time. MTIs have been introduced into oncological therapy only in recent years. Temsirolimus can be used for the treatment of metastatic renal cell carcinoma [24] and relapsed or refractory mantle cell lymphoma [25]. Significant preclinical evidence has been accumulated about mTOR inhibition as a feasible therapeutic strategy in several other types of human solid cancers [26] and in lymphoid malignancies [27], [28], including ALL [29], [30].

The mTOR pathway would therefore be an attractive therapeutical target in childhood ALL as well. In order for this, we need to find markers which help determine the activation level of the pathway, and could predict response to mTOR inhibitor therapy – possibly by using easily applicable, quantitative, routine diagnostic methods.

The aim of our study was to determine mTOR activity in childhood ALL cells (cell lines, and leukemia cells isolated at diagnosis and during treatment). mTOR activity was characterized by measuring the phosphorylated form of two mTOR dependent proteins (p-4EBP1 and p-S6). Differences in mTOR activity were compared to the immunophenotype of ALL (BCP- [B-cell progenitor-] or T-ALL) and clinical response to treatment (good and poor prognosis). mTOR activity was monitored during treatment, and we sought to determine its role in treatment response evaluation and as a prognostic factor. The effect of rapamycin treatment alone, and rapamycin combined with different chemotherapeutic agents was also investigated *in vitro* in ALL cell lines and in cells isolated from childhood ALL patients.

## Materials and Methods

### Ethics Statement

Samples were obtained with informed consent and all protocols were approved by the Institutional Ethical Review Board *(TUKEB – Scientific and Reserach Ethics Council, Hungary – no. 53/2011).* Written informed consent was obtained from the parents on behalf of the children participants involved in our study.

### Patients

Initially, peripheral blood and bone marrow samples (n = 269) were collected from children with ALL. Diagnosis was based on clinical findings, cytomorphology, flow cytometry, cytogenetics and molecular genetic analyses of blasts, according to the treatment protocol. Some of the samples were inadequate for measurements and statistical analysis because of the low number of isolated lymphoblasts. Samples from all phases of treatment (collected at diagnosis, and at conventional response checkpoints [at days 15, 33, and 3 months after the beginning of therapy]) were available from 21 patients; additional samples were analysed at diagnosis from 28 other patients (from these patients, samples were obtained at relapse, where applicable), and from one patient only at relapse. Bone marrow samples from 42 children with BCP-ALL, from 8 children with T-ALL, and from non-leukemic patients were used for further examination. Five patients died during the follow up period: four patients due to the progression of the disease, and one patient due to transplant related toxicity.

Eventually, appropriate samples from 49 children (15 females and 34 males) with primary ALL were examined and data was subjected to statistical analysis. Average age at diagnosis was 6.6 years (range 1.8–16.4 years); 59% of the patients belonged to the group with favorable prognosis, based on their age (6 years or less). An initial WBC count of 20 G/l is the cutoff level for defining different prognostic groups according to our treatment protocol; based on this, 57% of the patients had better prognosis, with the initial WBC count being less than 20 G/l.

Cytogenetic and molecular genetic karyotyping of the blasts revealed the following alterations: a. 2 patients possessed a Philadelphia chromosome (t[9]; [22], BCR/ABL); ABL amplification was detected in one patient, and RUNX1 amplification was seen in one patient – these genetic lesions indicate an unfavorable prognosis for these patients, all of whom (n = 4) were treated according to the high risk (HR) arm of the protocol; b. 19 patients with hiperdiploidy and two patients with ETV6/RUNX1 fusion were assigned to the group with considerably good prognosis (termed hyperdiploid group; n = 21); c. karyotyping revealed genetic lesions irrelevant to prognosis, or no genetic alterations in 25 patients (termed normal karyotype group; n = 25).

Lymphoblasts derived from bone marrow and peripheral blood were both used and compared to each other in experiments in order to define mTOR signaling pathway activity. Clinical data were collected prospectively from the date of diagnosis. All relevant clinical and laboratory data of the 49 patients are summarized in Table 1. Most ALL patients were treated according to the ALL IC-BFM 2002, two patients according to the ALL IC BFM 2009, one patient according to the ALL BFM 95 protocol. According to the prognostic factors described in treatment protocols, patients received different intensities of therapy: 21 (42%) children were treated in the standard risk arm (SR), 19 (39%) in the intermediate risk arm (IR) and 9 (19%) in the high risk arm (HR) of the protocol.

**Table 1:** Summary of clinical data and mTOR activity related p-4EBP1 in 49 primary ALL patients.

		low mTOR activity§	high mTOR activity§	P value
	number (%)	number (%)	number (%)	
**Total**	49 (100%)	39 (79%)	11 (21%)	
**Age**				
6 years or less	29 (59%)	23 (79%)	6 (21%)	0.722χ
6 years <	20 (41%)	15 (75%)	5 (25%)	
**Gender**				
female	15 (31%)	14 (93%)	1 (7%)	
male	34 (69%)	24 (71%)	10 (29%)	0.137φ
**Cell type**				
pre-T	8 (16%)	4(50%)	4 (50%)	
pre-B	41 (84%)	34 (83%)	7 (17%)	0.063φ
**Steroid response,** π				
response at day 8	40 (81%)	35 (88%)	5 (12%)	
no response at day 8	9 (19%)	3 (33%)	6 (67%)	<<0.05φ
**WBC count**				
20 G/l>	21 (43%)	17 (81%)	4 (19%)	
20 G/l<	28 (57%)	21 (75%)	7 (25%)	0.737φ
**Karyotype** * **^,^** π				
normal	24 (49%)	24 (71%)	7 (29%)	
hyperdiploid	21 (43%)	20 (95%)	1 (5%)	<0.05χ
high risk genetic lesion	4 (8%)	1 (25%)	3 (75%)	
**Prognosis** #,π				
good	37 (76%)	33 (89%)	4 (11%)	<<0.05φ
poor	12 (24%)	4(33%)	8 (67%)	
**Present status** π				
disease free remission in				
SR group	21 (43%)	20 (95%)	1 (5%)	
IR group	16 (33%)	13 (81%)	3 (19%)	
HR group	5 (10%)	3 (60%)	2 (40%)	<<0.05χ
remission (after relapse or TX)	3 (6%) - 2 IR; 1HR	2 (67%)	1 (33%)	
death (after progression or TRM)	4 (8%) - 1 IR; 3HR	0 (0%)	4 (100%)	

§ cutoff equals 1.1 (OD p-4EBP1 ELISA).

* based on cytogenetic and molecular genetic analysis; the hyperdiploid group also includes two cases with ETV6/RUNX1 fusion (good prognosis). High risk genetic lesions are: BCR/ABL fusion (two cases), ABL amplification (one case) and RUNX1 amplification (one case).

# based on steroid response and current status; see methods: SR - standard, IR - intermediate, HR - high risk arm of the therapy; TX – transplantation, TRM - transplantation related mortality.

φ – Fisher’s exact probability and,

χ - chi^2^ tests.

π - significant correlation with mTOR activity level.

Prognosis was evaluated in all 49 patients accordingly, based on: 1. WBC count at diagnosis, age, prednisolone response at day 8 (absolute blast cell count in peripheral blood), bone marrow blast percentage on day 15 and on day 33; and 2. relapse during long-term follow-up – patients with good prognosis were in remission during the observation period, and some patients with initially good prognosis relapsed or progressed during this time period, regardless of evaluation based on criteria in point 1. Three such patients were initially in the good prognostic group, but relapsed during follow-up. Therefore, these patients were reassigned later to the poor prognostic group, such that the final number of patients in this group was 12 at statistical analysis. The samples and data of patients were collected in the time period of 2006–2012.

### Primary ALL Cells, Cell Lines and Culture Conditions

Lymphoblasts were isolated from bone marrow and peripheral blood samples of childhood ALL patients and healthy/non-leukemic individuals by ficoll gradient centrifugation (Histopaque 1077, Sigma Aldrich, St. Louis, MO). Normal B-cells and T-cells were obtained by MACS CD19 micromagnetic beads (Miltenyi Biotec, Auburn, CA) and by nylon wool fiber (Wako Chemicals), respectively. Isolated cells were stored at −70°C or short-term cultured.

Previously characterized human ALL cell lines (Nalm6– B-cell precursor leukemia, clone ACC 128, German Tissue Collection of Microorganisms and Cell Culture [DSMZ]; Mn60– B-cell leukemia established from acute B-ALL, clone ACC138, DSMZ; Jurkat – acute T cell leukemia, clone E6-1, American Type Culture Collection [ATCC]; CCRF-CEM –acute T lymphoblastic leukemia, clone #82112105, European Collection of Cell Culture [ECACC]); and a Hodgkin-lymphoma cell line (KMH2, clone ACC8, DSMZ) were used for *in vitro* experiments.

Lymphoblasts and cell lines were cultured in RPMI 1640 Medium (Sigma), supplemented with 10–20% fetal calf serum (FCS, Sigma), 0.03% L-glutamine and antibiotics (Sigma) at 37°C in a 5% CO_2_ humidified atmosphere. Cells were cultured on 24-well plates or in 25 cm^2^ flasks (2–4×10^5^ cells/ml), and treated for 0–72 hours with the following chemotherapeutic agents: rapamycin (Rapamycin, Sigma), methotrexate (Methotrexate, Teva, Pharmachemie BV), cytosine arabinoside (Alexan, Ebewe Pharma), doxorubicin (Doxorubicin, Ebewe Pharma), vincristine (Vincristin liquid, Richter Gedeon), etoposide (Etoposide, Teva, Pharmachemie BV), methylprednisolone (Methyl-prednisolon-Human, TEVA) and cyclophosphamide (Endoxan, Baxter Oncology). Each cell line was cultured and exposed to drugs in triplicates in at least 3 independent experiments. Cell morphology was evaluated on methanol fixed and hematoxylin-eosin (HE) stained cytospin preparates (5′ 500 rpm).

### Apoptosis Detection by Flow Cytometry

Apoptosis measurements were performed according to Mihalik, et al [31]. Briefly, cells were fixed in 70% ethanol (at −20°C), followed by alkalic extraction (200 mM Na2HPO4, pH 7.4), addition of 100 µg/mL RNase (Sigma) and propidium iodide staining (95–98%, Sigma). Membrane permeabilization and alkalic extraction allow the release of small molecular weight DNA fragments from apoptotic cells, and the apoptotic population becomes easily detectable as the subG1 fraction on histograms [32]. Apoptosis measurements were performed on a FACSCalibur flow cytometer (BD Biosciences) using Cell Quest software (BD Biosciences); 10–20 000 events were collected for each sample. Data was analyzed with WinList software (Verity Software House).

### ELISA Analysis

Cell lysates were obtained from isolated bone marrow or peripheral blood derived ALL cells, isolated normal lymphoid cells and cell lines, in Cell Lysis Buffer (Cell Signaling) containing 1 mM phenyl-methylsulfonyl fluoride (PMSF) for 30 minutes on ice (1 M cells/sample/100 µl Lysis Buffer). Protein concentration was determined by Qubit® fluorometry (Invitrogen) to ensure equal cell numbers. Samples with significantly lower or higher protein concentrations were not involved in this study; mean protein concentration of analyzed samples was 16.4±0.15 µg/ml. Sandwich ELISA Kits (PathScan p-4EBP1 - Thr37/Thr46 and PathScan p-S6 Ribosomal Protein - Ser235/236, Cell Signaling) were used for the detection of p-4EBP1 and p-S6 ribosomal protein, according to the manufacturer. Absorption and optical density (OD) were measured at 450 nm wavelength. A p-4EBP1 and p-S6 overexpressing cell line (KMH2) was used as a positive control.

Several methods were tested to investigate the amount of mTOR kinase activity related proteins in blood and bone marrow samples of ALL patients. Different ELISA kits requiring lower cell numbers were tested to analyze and compare various samples. The p-4EBP1 ELISA kit mentioned above was the most reliable one. Data obtained with this assay correlated well with Western blotting results, and showed reduced mTOR activity after in vitro rapamycin treatment in cell lines (data not shown). Moreover, this assay was more sensitive than p-S6 ELISA, even though p-S6 is the most commonly used marker for mTOR activity in immunohistochemistry.

### Immunocytochemistry

Cytospin preparates were incubated with primary antibodies (anti-phospho-S6 and anti-phospho-mTOR, 1∶100 and 1∶50, respectively; Cell Signaling) after endogenous peroxidase blocking. Immunodetection was performed by Novolink detection system (Novocastra), visualized by DAB (Dako) and counterstained with haematoxylin. p-4EBP1 and p-S6 immunostaining was also performed in liquid phase and measured by flow cytometry: 2 M cells/sample were labeled with 2 µl anti-phospho-S6 or anti-p-4EBP1 antibody (Cell Signaling), followed by biotinylated anti-rabbit IgG secondary antibody (Vector Laboratories) and Qdot 705 streptavidin conjugate (Invitrogen). Measurements were performed on a FACSCalibur flow cytometer (BD Biosciences) with CellQuest Pro software; 10–20 000 events were collected for each sample. The differences between mean fluorescence intensities (MFI) of unstained negative control cells, and samples stained with anti-p4EBP1 or p-S6 analyzed in parallel were calculated using CellQuest Pro software (BD Biosciences).

### Statistical Analysis

Mean arithmetic values (x) and standard deviation (SD) of the data were calculated. Variables were compared with Student’s t-test if sample distribution was normal, or Mann-Whitney U test if sample distribution was asymmetrical. The cutoff point for ELISA OD – which segregates patients into good and poor prognostic groups with appropriate specificity and sensitivity – was calculated by ROC curve analysis. Categorical data were compared using chi^2^ test and Fisher’s exact probability test to evaluate significance. Overall survival analyses were done using the Kaplan-Meier method. Survival intervals were determined as the time period from initial diagnosis to the time of death (overall survival) or relapse (relapse free survival). Survivals of groups stratified by p-4EBP1 levels – indicating mTOR activity – was compared with the log-rank test. Multivariate analysis of different prognostic factors was done using the Cox regression model.

P<0.05 was considered statistically significant. Statistical analysis was performed with Statistica 9.0 (Statsoft, Tulsa, OK) and SPSS software.

## Results

### P-S6 and p-4EBP1 are Over-expressed in Leukemia Cells

**Figure 1:**
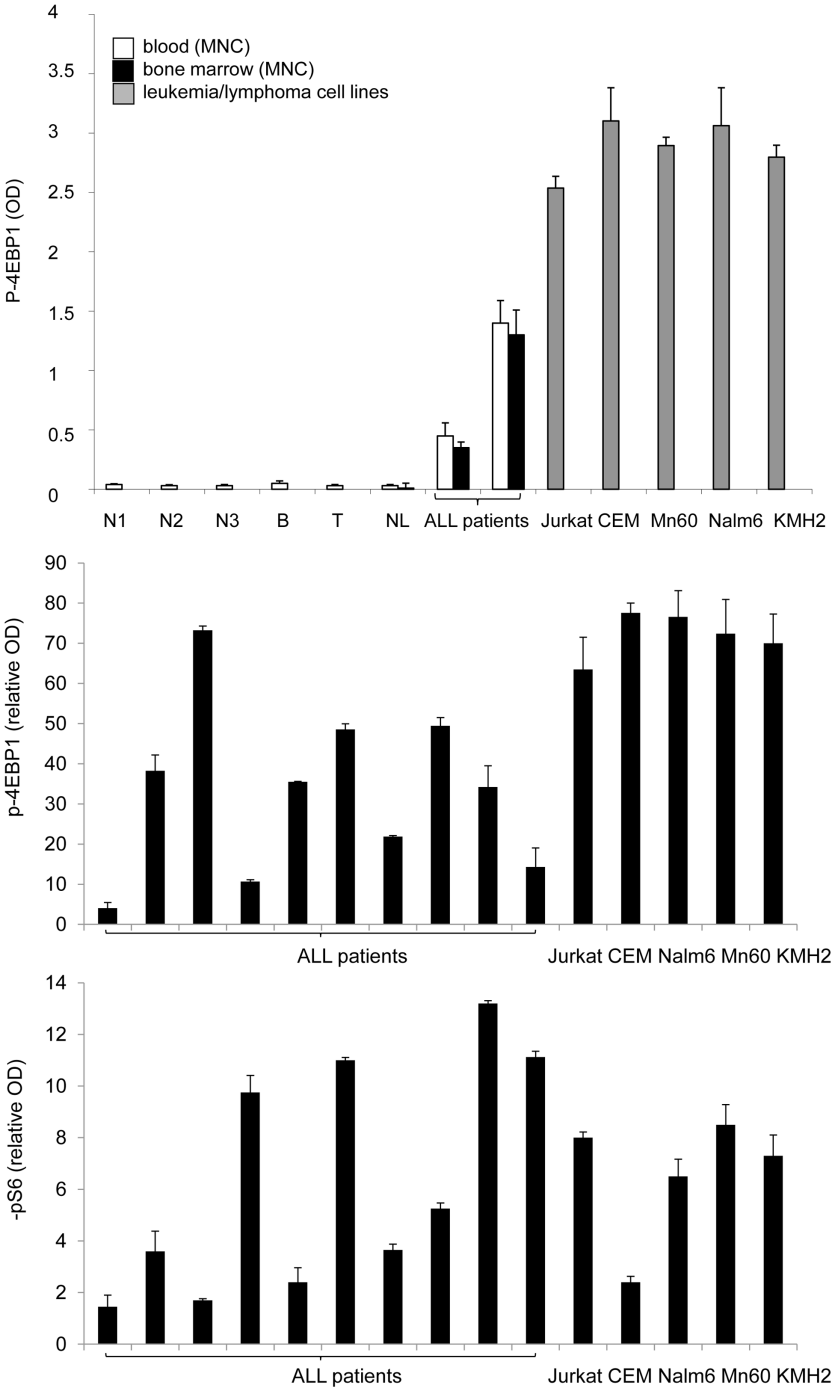
The amount of mTOR activity dependent phospho-proteins (p-4EBP1 and p-S6) in lymphoma/leukemia cells (ELISA). (a.) P-4EBP1 ODs are shown for normal control peripheral blood mononuclear cells (N1, N2, N3), isolated peripheral normal B- and T-cells (B, T), isolated peripheral blood mononuclear cells and isolated bone marrow mononuclear cells from non-leukemic patients (NL) and isolated primary childhood ALL cells from representative patient samples, and from leukemia (Jurkat, CEM, Mn60, Nalm6) and lymphoma cell lines (KMH2). (b.) Relative p-4EBP1 and p-S6 expression was determined in samples from ALL patients (n = 10) and in leukemia/lymphoma cell lines (Jurkat, CEM, Mn60, Nalm6, KMH2); expression of normal lymphoid cells is considered to be 1. (OD: optical density; MNC: mononuclear cells. Equal cell numbers and equal protein concentrations were confirmed for comparisons.).

**Figure 2:**
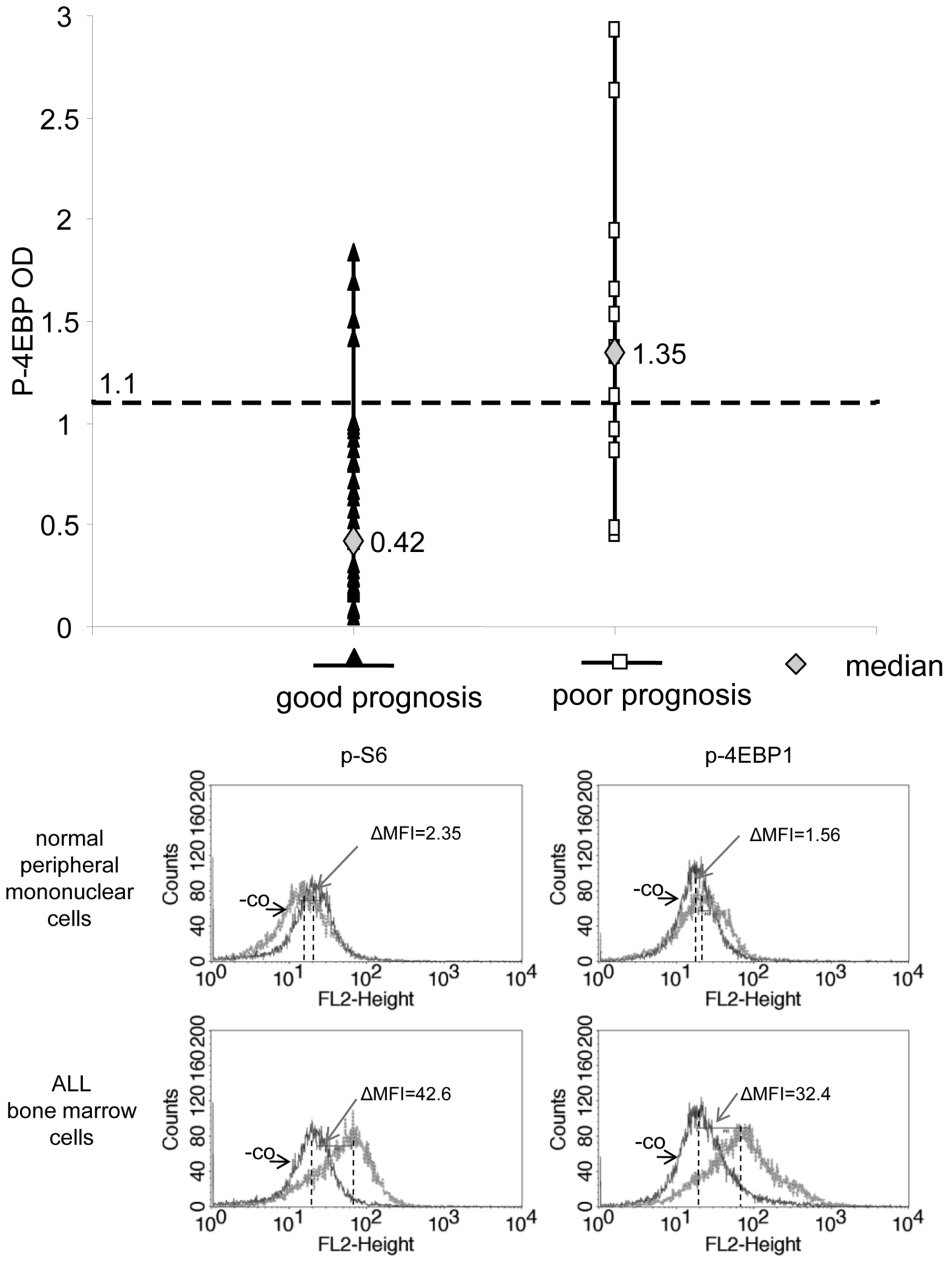
mTOR activity related p-4EBP1 expression in samples of ALL patients with different prognoses (ELISA) and detection of p-4EBP1 and p-S6 in ALL cells by flow cytometry. (a) P-4EBP1 OD values in 49 ALL samples (n = 37 for patients with good prognosis; n = 12 for poor prognosis, criteria for prognostic groups are described in the Methods section). P-4EBP1 OD levels were significantly different between patients with good and poor prognosis (p<0.05). The cutoff value between p-4EBP1 OD values representing high and low mTOR activity was determined to be 1.1 by ROC curve analysis. (b) p-S6 and p-4EBP1 overexpression is confirmed by flow cytometry in ALL cells. Representative histograms for p-4EBP1 and p-S6 flow cytometric analysis in normal PMNC and in ALL samples. Expression was calculated as the difference between MFIs of p-4EBP1 or p-S6 stained (p-4EBP1 and p-S6, respectively) and parallel unstained controls (-co). Differences between MFIs (ΔMFI) was 2.35 (pS6) and 1.56 (p-4EBP1) for normal peripheral mononuclear cells. However, ΔMFI was 42.6 and 32.4 for p-S6 and p-4EBP1, respectively, in the representative ALL case shown here.

The expression of the mTOR pathway targets p-S6 and p-4EBP1 was characterized in human acute lymphoblastic leukemia cells and normal lymphoid cells by ELISA. (The KMH2 cell line was used as a positive control [p-4EBP1 OD: 2.8±0.01; pS6 OD: 0.37±0.02].) The value for normal lymphoid cells was considered to be 100% (or 1) for comparisons. The amount of activated mTOR targets was not significantly different in peripheral blood mononuclear cells (PMNC) and bone marrow mononuclear cells (BMMNC) within the same healthy individuals or ALL patients investigated (Fig. 1a). Leukemia and lymphoma cell lines (Nalm6, Mn60, Jurkat, CEM and KMH2) showed increased p-S6 (2.4 to 8.5-fold) and remarkably elevated p-4EBP1 (63.5 to 77.6-fold) levels by ELISA, compared to normal lymphoid cells (isolated PMNC, T- and B-cells). Similarly to cell lines, p-4EBP1 and p-S6 expression was significantly elevated in ALL samples (both PMNC and BMMNC) at diagnosis, compared to normal lymphoid cells (Fig. 1b, Fig. 2a). However, upregulation of pS6 was less pronounced.

P-4EBP1 and/or p-S6 levels were also analyzed by immunocytochemistry on cytospin slides (n = 7) and by flow cytometry (n = 3) in ALL samples. Elevated mTOR activity was detected in ALL blasts with both stainings, compared to normal peripheral mononuclear cells. ALL cells showed moderate or strong expression, evaluated by two independent cytopathologists; however, normal cells were scored negative or only weak expression was detected on cytospin slides (Fig S1). Flow cytometric analysis also revealed high p-4EBP1 and p-S6 staining in all three examined ALL samples: mean fluorescent intensity (ΔMFI) was 15–20 fold compared to non-leukemic control samples (Fig. 2b).

### Correlation of p-4EBP1 Expression at Diagnosis with Prognosis/Response to Therapy and other Clinical Data

The expression level of p-4EBP1 was analyzed at day 0 in all primary samples from the 49 different ALL patients. P-4EBP1 level was higher in all ALL samples than in normal PMNC, isolated B or T-cells. The amount of p-4EBP1 detected by ELISA was significantly higher at day 0 in the BMMNCs of ALL patients with poor prognosis – both in B- and T-ALL patients. We established a cutoff point of OD = 1.1 for p-4EBP1 ELISA values, which segregates ALL patients into good (OD<1.1) and poor (OD>1.1) prognostic groups (Fig. 2a). The cutoff point with appropriate specificity (89%) and sensitivity (67%) was calculated with ROC curve analysis. Samples with p-4EBP1 OD values higher than 1.1 were considered to have high mTOR activity, whereas OD<1.1 was considered to represent low mTOR activity. An OD of 1.1 equals a 27,5-fold increase in p-4EBP1 levels compared to normal lymphoid cells.

**Figure 3:**
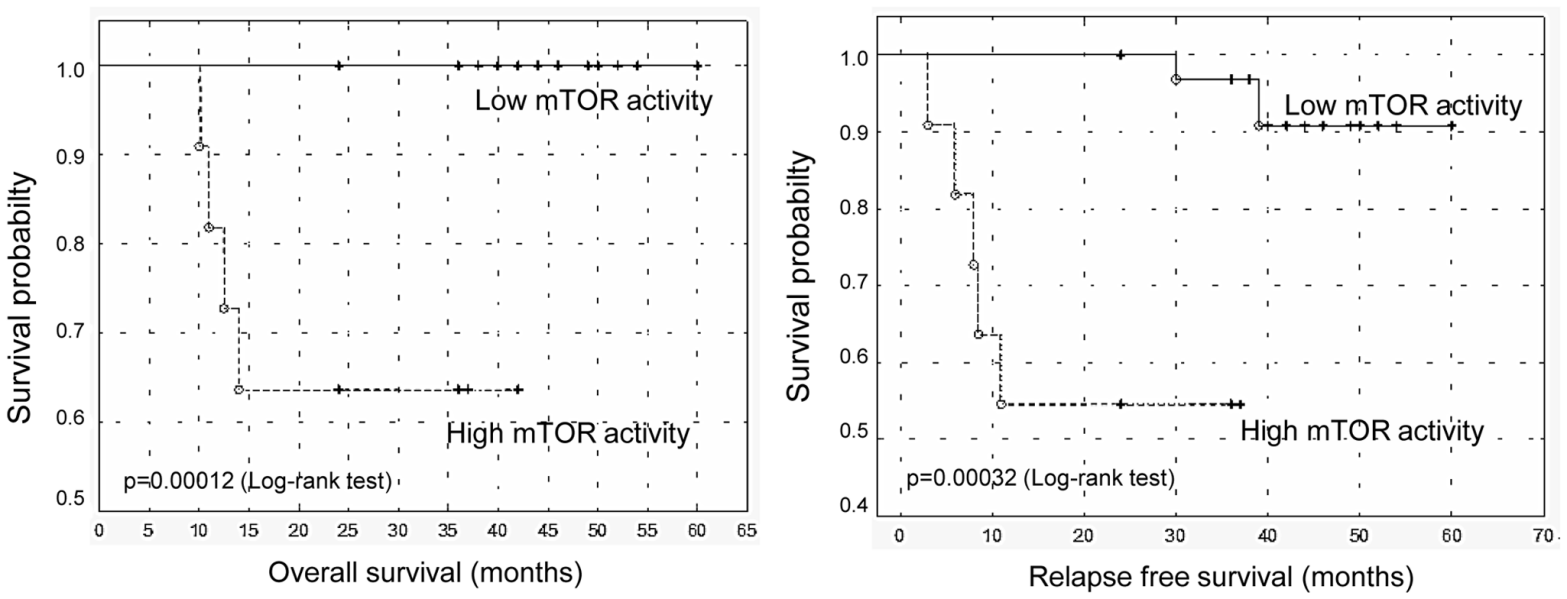
Survival analysis (Kaplan-Meier curves). Kaplan-Meier curves show overall and relapse free survivals for patients (n = 49) stratified by mTOR activity. Cutoff value between low and high mTOR activity groups (n = 37 and n = 12, respectively) was determined to be 1.1 for p-4EBP1 ODs (ELISA). We found that patients with low mTOR activity had significantly longer overall and relapse free survival (P<<0.05 with log-rank test; o: complete event, +: censored cases).

Significant statistical correlation was found between poor prognosis (12 patients), poor steroid response at day 8 (9 patients) and high levels of mTOR activity related p-4EBP1. Moreover, low p-4EBP1 levels showed significant correlation with relapse free survival (present status). A significant correlation between non hyperdiploid karyotypes and high mTOR activity was also established. However, no significant association between mTOR activity and other clinical data (age, gender, cell type and WBC count) was detected by statistical analysis (Table 1). Next, we used the Kaplan-Meier method to analyze survival data stratified by p-4EBP1 levels (indicative of mTOR activity), and we showed a significant difference in overall survival and relapse free survival between patients with low and high mTOR activity (Fig. 3). Multivariate analysis (including standard prognostic variables, such as age, gender, cell lineage and WBC count) also indicated that high levels of p-4EBP1 detected by ELISA (>1.1 OD) predicted poor outcome, i.e. it increased relative risk for relapse/poor therapeutic response, independently of other variables (Table 2).

**Table 2:** Multivariate analysis of various standard independent prognostic factors in ALL cases.

Prognostic factors	RR (95% CI)	p
Age in year (<6 versus >6)	0.204 (0.025–1.65)	0.136
Gender (female versus male)	na	1
Cell type (B versus T)	5.148 (0.429–61.8)	0.196
WBC (<20 G/l versus >20 G/l)	0.249 (0.025–2.48)	0.236
mTOR activity (low versus high)π	37.07 (2.552–538.51)	0.0081

* Cutoff value is 1.1 p-4EBP1 OD.

RR – relative risk, 95% CI –95% confidence interval.

na – not applicable.

**Figure 4:**
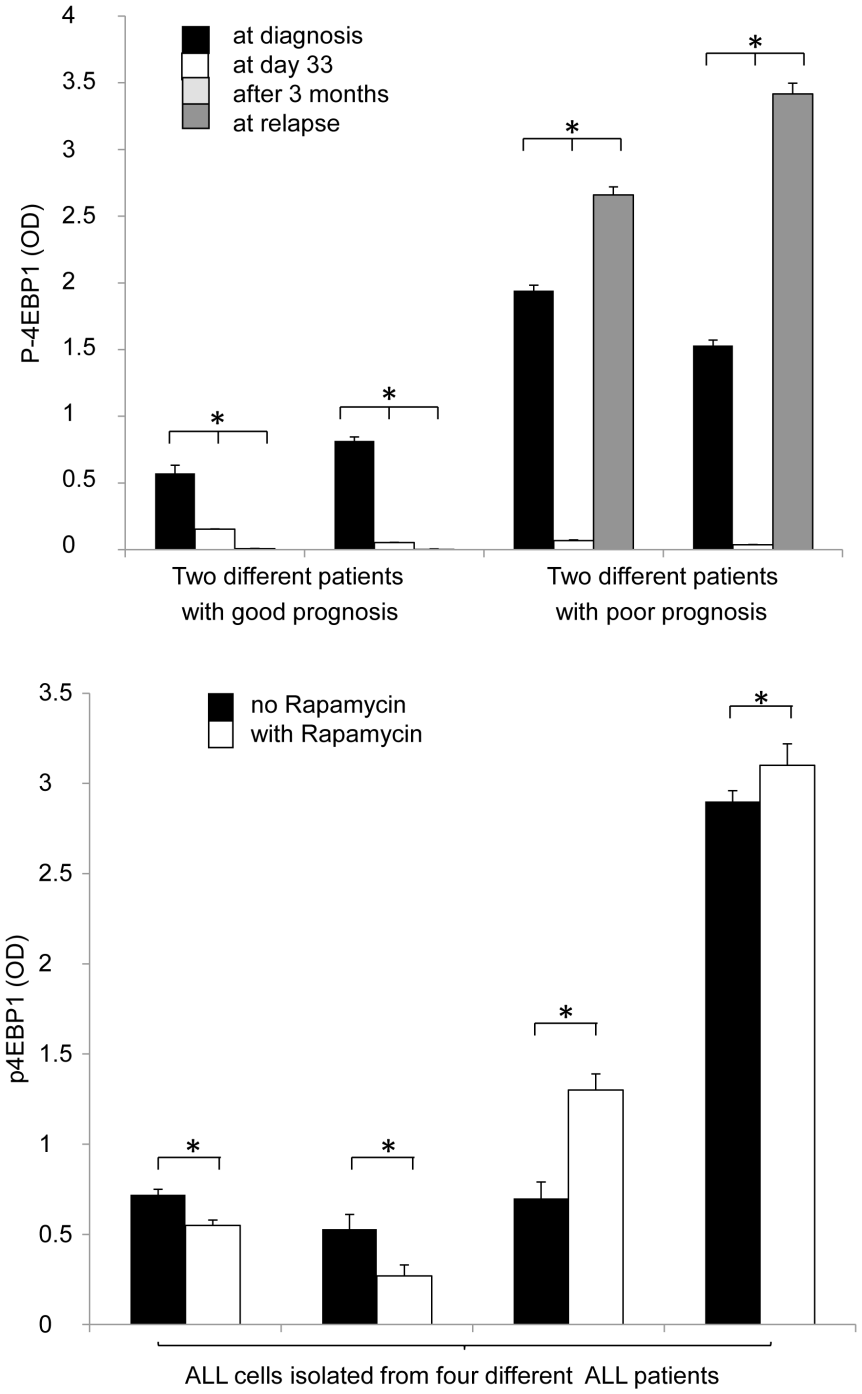
Changes in mTOR related p-4EBP1 expression in samples of ALL patients after *in vivo* chemotherapy and in cultured ALL cells treated with rapamycin *in vitro*. (a) P-4EBP1 OD is reduced by chemotherapy, but re-increases during relapse in ALL patient samples. Data is shown from 2 representative patients with good prognosis and 2 patients with poor prognosis at day 0, day 33, after three months of treatment, and at relapse (ELISA). (b) *In vitro* rapamycin sensitivity is variable in different human ALL samples (n = 4). P-4EBP1 levels variably decrease or increase after *in vitro* rapamycin treatment (50 ng/ml, 24 h) in isolated ALL cells (ELISA). A decrease in p-4EBP1 was associated with concomitant induction of apoptosis by rapamycin (>50% apoptotic cells in the first two samples; data not shown). (The first three samples were cultured from cells isolated from primary ALL cases and one at relapse.) *- P<0.05.

Samples from 21 patients were available for analysis at diagnosis and during treatment follow-up, which enabled us to monitor expression changes during therapy and at relapse. P-4EBP1 expression significantly decreased during treatment in all 21 examined ALL cases, with a concomitant reduction in the percentage of lymphoblasts (0–10% of bone marrow mononuclear cells). P-4EBP1 expression was reduced in patients with good prognosis (OD<1.1) after treatment (compared to day 0), and remained so after two years. However, in the two relapsed patients examined, we detected high p-4EBP1 expression (OD>1.1) at day 0, which predicted unfavorable prognosis, and this was clinically confirmed later; in these cases, the expression of p-4EBP1 protein decreased significantly at day 33, but increased above the initial level at relapse (Fig. 4a).

### Rapamycin Modifies Apoptosis and p-4EBP1 Expression in ALL Short-term Cultures

Leukemia cell lines and lymphoblasts obtained from bone marrow samples of ALL patients were kept in short-term culture and treated with rapamycin (50 ng/ml). Rapamycin monotreatment had only an antiproliferative effect with no apoptosis induction in Nalm6, Mn60 and CEM cells; however, it induced apoptosis in Jurkat cells after 72 h treatment (data not shown).

Baseline spontaneous apoptosis was higher in short-term cultures of primary ALL cells (approaching 30% after 24 h) than in cell lines; moreover, rapamycin was able to increase apoptosis significantly in short-term cultures after 24 h treatment in some, but not all primary isolated ALL lymphoblast cultures. Apoptosis induction was accompanied by reduced p-4EBP1 expression after rapamycin treatment. We were able to analyze the apoptotic effect and p-4EBP1 expression in parallel in four samples treated with rapamycin for 24 h. Rapamycin did not decrease the expression of p-4EBP1 in two cases (one isolated from primary ALL and one at relapse), and apoptosis induction was not significant (<10% increase in apoptosis). However, rapamycin treatment enhanced apoptosis significantly (>50% apoptotic cells) in the other two cases (isolated from primary ALL), accompanied by a concomitant reduction of p-4EBP1, which was also significant (Fig. 4b).

### Rapamycin Enhanced the Apoptotic Effect of Chemotherapeutic Agents in Leukemia Cell Lines and in Short-term ALL Cultures

**Figure 5:**
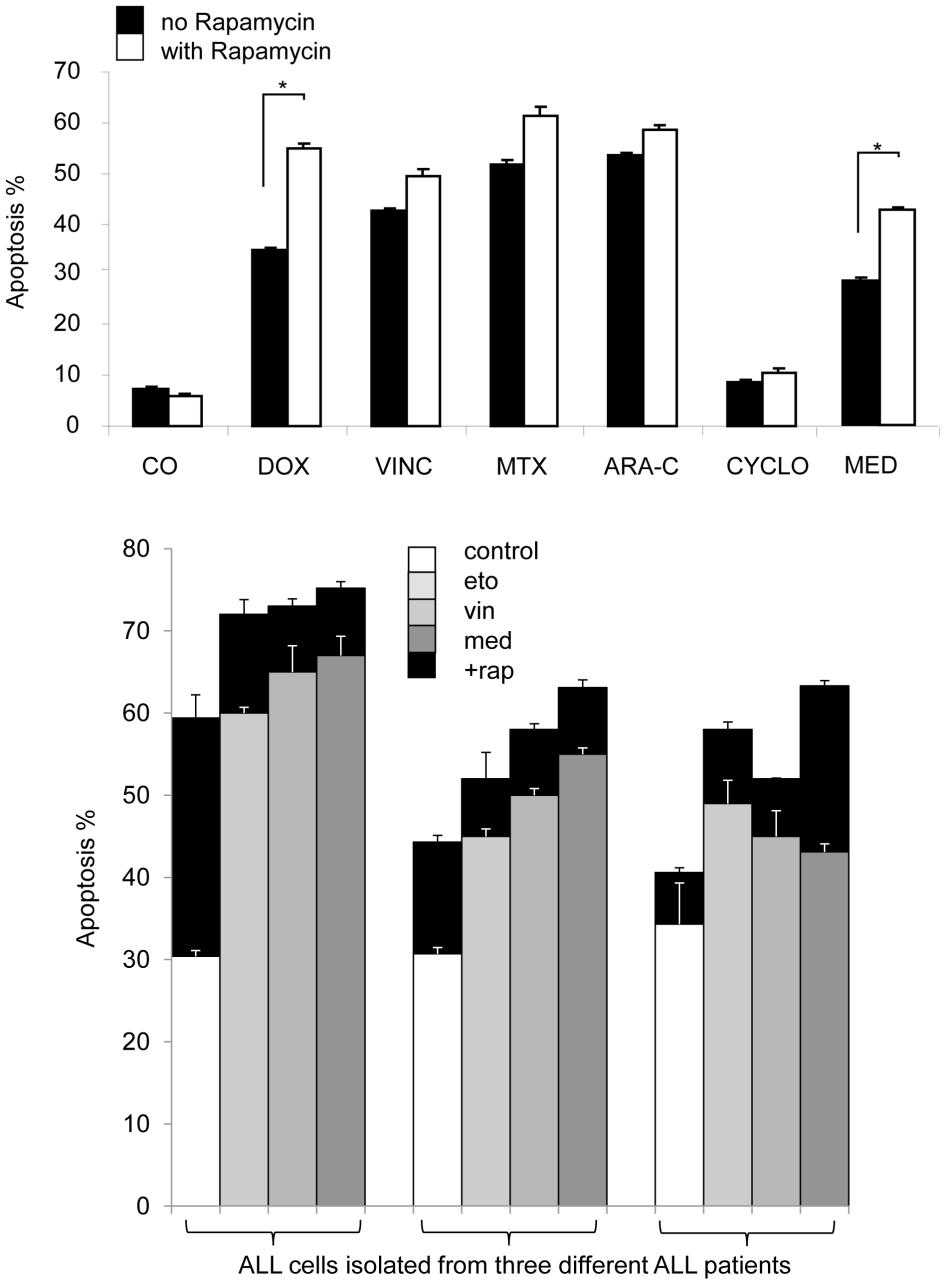
Rapamycin increases the apoptotic effect of several chemotherapeutics in leukemia cells *in vitro* (flow cytometry). (a) The apoptotic effect of rapamycin (50 ng/ml) combined with other drugs. Nalm6 cells were treated for 72 h. (DOX: doxorubicin, 100 nM; VINC: vincristin, 3 nM; MTX: methotrexate, 20 nM; ARA-C: cytarabine, 10 ng/ml, CYCLO: cyclophosphamide, 500 nM; MED: methyl-prednisolone, 100 nM.) *- p<0,05**.** (b) The apoptotic effect of rapamycin (rap, 50 ng/ml) combined with methyl-prednisolone (med; 100 nM), vincristin (vin; 3 nM) and etoposide (eto; 10 nM) treatment in isolated childhood BCP-ALL cells from three different patients, treated for 24 h. (The first two samples were cultured from cells isolated at diagnosis, the third one at relapse).

Cell lines and lymphoblasts isolated from 3 primary and 2 relapsed ALL patients were treated with rapamycin combined with chemotherapeutics (doxorubicin, etoposide, vincristine, methotrexate and methyl-prednisolone). Rapamycin increased the apoptotic effect of all tested chemotherapeutics in Nalm6 (Fig. 5a) and Jurkat cells. However, Mn60 and CEM cells were less sensitive to combination treatment. Rapamycin enhanced apoptosis induced by cytosine arabinoside, etoposide and methyl-prednisolone in Mn60 cell cultures, whereas it promoted only the effect of methyl-prednisolone in CEM cells in vitro.

The effect of rapamycin and chemotherapeutic drugs was variable in BMMNC isolated from ALL samples. In two patients (one primary and one relapsed), induction of apoptosis was not significant (<l0%) by rapamycin and the combinations. In other three cases (2 primary cases and one relapsed), apoptosis induction by rapamycin was significant (22–94%), and rapamycin enhanced the effect of etoposide, vincristine and methyl-prednisolone (Fig. 5b).

## Discussion

Increased mTOR activity has already been reported and data has been published about the *in vitro* apoptotic effects of rapamycin in cell lines and isolated primary ALL cells [29], [33], [34]. However, quantitative analysis of mTOR pathway activity in childhood ALL patients is lacking. Furthermore, mTOR activity and the amount of related phosphoproteins has not been determined by ELISA in human leukemia cells; only one study describes an ELISA-based method for measuring p-p70S6K as an assessment of mTOR inhibition in peripheral lymphoid cells from renal allograft recipients treated with everolimus [35].

In the present study we investigated the activity of the mTOR pathway by ELISA in bone barrow samples from childhood ALL patients. The levels of phosphoproteins (p-4EBP1 and p-S6) related to mTOR activity were determined in normal lymphoid cells and in isolated lymphoblasts. Several ELISA kits were tested, and we found that the p-4EBP1 and the p-S6 assays worked reliably in our study, with the p-4EBP1 kit being the most useful. Measuring p-4EBP1 is also advantageous because 4EBP1 is directly phosphorylated – and thus inhibited – by mTOR. In contrast, S6 protein can be phosphorylated not only through the mTOR pathway – by p70S6 kinase –, but also by other signaling pathways, such as PDK1, MAPKs and stress activated protein kinase (SAPK) [36].

We found that mTOR signaling pathway activity (indicated by p-4EBP1 levels) was high in leukemia/lymphoma cell lines and in bone marrow cells from ALL patients at day 0. The level of phosphorylated mTOR target proteins – p-4EBP1 and p-S6– fell back to virtually normal in ALL samples upon response to chemotherapeutic treatment. In samples of relapsed patients, mTOR activity increased when the disease reappeared: indeed, p-4EBP1 expression was above day 0 levels at relapses. Interestingly, mTOR activity was significantly higher at diagnosis in patients with poor prognosis than in patients with good prognosis. Using ROC curve analysis, we established a cutoff point (OD = 1.1) for p-4EBP1 ELISA values, which segregated patients into two groups: one with high p-4EBP1 levels, and consequently, high mTOR activity, and another group with low mTOR activity. This cutoff value can be validated in larger studies, and may serve as a clinically available prognostic marker for ALL in the future. The disadvantage of ELISA is that it is time-consuming and requires relatively large numbers of cells. A more easily applicable method, such as flow cytometry, might be more useful for the detection of mTOR activity related phosphoprotein expression at diagnosis or during follow-up.

It is an important observation that mTOR activity – characterized by p-4EBP1 expression – is not significantly different in bone marrow and peripheral lymphoid cells from the same patient. Patients with a greater chance of relapse – who, therefore, require closer follow-up – may be monitored by measuring mTOR activity in peripheral blood samples. Several studies focus on the issue of early detection of disease recurrence, however, an effective method – which is minimally invasive and shows relapse very early – does not exist yet.

Several studies have been initiated to investigate the effects of MTI treatment in childhood ALL [29], [8], [37], and our *in vitro* experiments also confirm the ability of mTOR inhibitors to induce apoptosis in isolated childhood ALL lymphoblasts. Many clinical trials have been launched to test the efficacy of mTOR inhibitors as single agents, particularly in the treatment of relapsed hematological diseases (eg. everolimus for relapsed/refractory NHL – phase I; sirolimus for relapsed/refractory ALL/NHL – phase I) [33]. However, the results of clinical trials in childhood ALL have not yet been published. Only one abstract reports the results of a phase I trial with sirolimus in pediatric patients with relapsed/refractory leukemia. One paper describes the safety of sirolimus added to graft versus host prophylaxis after allogeneic hematopoietic stem cell transplantation in childhood ALL in a phase I/II trial [38]. Another paper reports maximum-tolerated dose (MTD), dose-limiting toxicities (DLTs), and pharmacokinetic and pharmacodynamic properties of everolimus in children with refractory or recurrent solid tumors in a phase I trial [39].

Instead of focusing on the effect of mTOR inhibitors as single therapeutic agents, the combination of mTOR inhibitors and conventionally used chemotherapeutic drugs (such as dexamethasone, doxorubicin, etoposide, vincristine and asparaginase) is currently being investigated more closely [37], [40], [41], [42]. Combination treatments with mTOR inhibitors are much more effective in inducing apoptosis, which is also supported by our study; the combination with methyl-prednisolone being the most effective, not only in cell lines but also in ALL samples. ALL patients frequently develop steroid resistance at relapse, and sirolimus has been reported to restore steroid sensitivity in steroid-resistant ALL [43], [44]. Correspondingly, we found that methyl-prednisolone combined with rapamycin induced significant apoptosis in isolated lymphoblasts in one ALL case after relapse.

It is important to note that the combination of rapamycin and various cytotoxic drugs may be more effective *in vivo* than *in vitro*, which may be explained by the modifying effects of the microenvironment. Combination treatments are also being examined in several clinical trials, mainly in relapsed hematological malignancies (acute myeloid leukemias and high risk lymphomas) [33], [25], [24].

Our results raise an important question: what selection criteria should be used for identifying ALL patients who may benefit from MTI therapies? Should we decide based on resistance to chemotherapy, the occurrence of relapse, or high pre-treatment mTOR activity? We suggest that patients with poor prognosis may be identified by measuring p-4EBP1 levels at diagnosis; further markers will be required for predicting the potential response to rapamycin treatment for individual patients within this group. Indeed, it must be kept in mind that high basal mTORC1 activity does not necessarily mean that the patient will respond to MTIs [45]. Our results demonstrate that *in vitro* rapamycin treatment does not necessarily result in the decrease of mTOR activity in ALL cells in all cases. The background of resistance to MTIs is not yet clear, but an increase in mTORC2 activity, and a shift in the proportion of mTORC1/C2 complexes must be considered. Dual inhibitors (inhibiting both mTORC1 and mTORC2 complexes) may be a solution for mTOR inhibition in rapamycin resistant cases, and promising experimental results have been published about their effect in leukemia cells [46], [47].

We showed that the activity of mTOR signaling is increased in childhood ALL lymphoblasts, and the level of activity can be monitored by p-4EBP1 ELISA. This may prove a useful method for selecting patients with poor prognosis at an early phase of the treatment. Patients with lymphoblasts exceeding a given level of mTOR activity may require closer follow-up, due to a significantly increased risk of relapse. The impact of mTOR activity on the survival capacity of ALL cells was also confirmed in vitro, which could be exploited by combining mTOR inhibitors with other therapeutic modalities.

### Conclusions

We determined the amount of mTOR activity related phosphoproteins in childhood ALL patients by ELISA. We suggest that the presence of these proteins (p-4EBP1 and p-S6) may serve as an early marker of prognosis, as patients with poor prognosis showed significantly higher p-4EBP1 level at diagnosis than patients with good prognosis. We established a cutoff value for p-4EBP1 ELISA OD levels, which may help segregate patients into good and poor prognostic groups. Early screening may also identify high risk patients and provide targets for therapy. We also found that mTOR activity was increased at relapse, and the detection of this increase may help in designing treatment and follow-up.

Taken together, our results provide a basis for further *in vitro* and *in vivo* studies for determining exact selection criteria for childhood ALL patients who may benefit from MTI therapy.

## Supporting Information

Figure S1 High p-S6 expression is confirmed by immunocytochemistry in samples of ALL patients. Representative p-S6 staining in isolated bone marrow mononuclear cells of an ALL- and a non-leukemic patient (immunocytochemistry; 400x).
(TIF)
