# Supplementary figures and images for: Mammalian Target of Rapamycin (mTOR) Activity Dependent Phospho-Protein Expression in Childhood Acute Lymphoblastic Leukemia (ALL)
Source: PLoS One. 2013 Apr 3;8(4):e59335. doi: 10.1371/journal.pone.0059335 (PMC3616065; doi:10.1371/journal.pone.0059335)

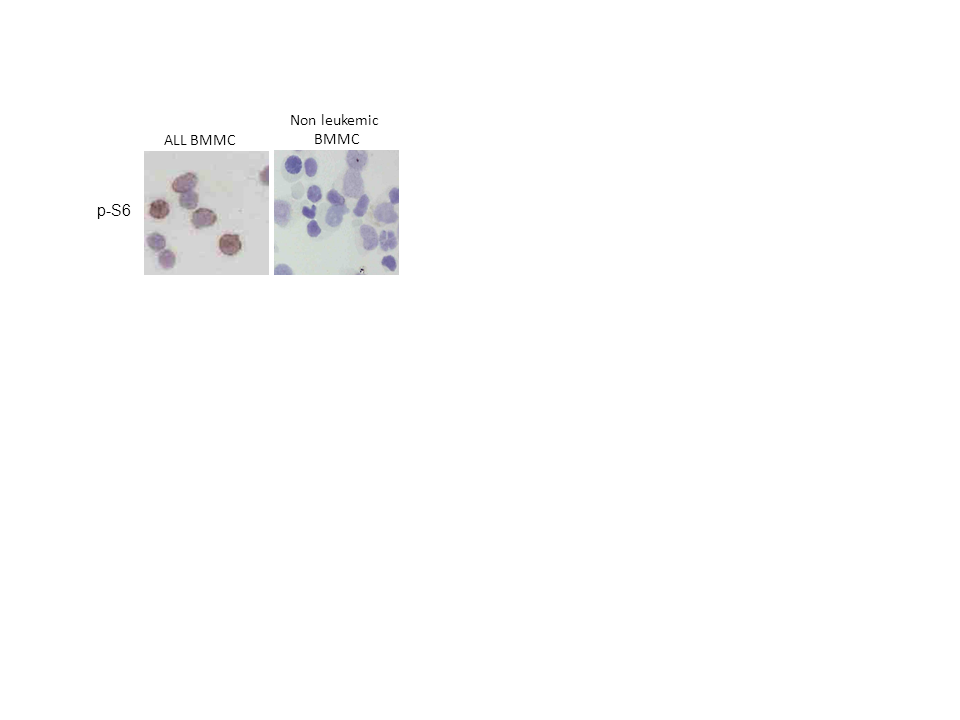

Supplement: Figure S1 — High p-S6 expression is confirmed by immunocytochemistry in samples of ALL patients. Representative p-S6 staining in isolated bone marrow mononuclear cells of an ALL- and a non-leukemic patient (immunocytochemistry; 400x). (TIF) [file pone.0059335.s001.tif]
